# Relative Pigment Composition and Remote Sensing Reflectance of Caribbean Shallow-Water Corals

**DOI:** 10.1371/journal.pone.0143709

**Published:** 2015-11-30

**Authors:** Juan L. Torres-Pérez, Liane S. Guild, Roy A. Armstrong, Jorge Corredor, Anabella Zuluaga-Montero, Ramón Polanco

**Affiliations:** 1 Bay Area Environmental Research Institute/NASA Ames Research Center, MS 245-4, Bldg 245, Rm. 120, Moffett Field, CA, 94035, United States of America; 2 Earth Science Division, NASA Ames Research Center, MS 245-4, Bldg 245, Rm. 120, P.O. Box 1, Moffett Field, CA, 94035, United States of America; 3 Bio-optical Oceanography Laboratory, Department of Marine Sciences, University of Puerto Rico, Mayaguez, Puerto Rico, 00680, United States of America; 4 Chemical Oceanography Laboratory, Department of Marine Sciences, University of Puerto Rico, Mayaguez, Puerto Rico, 00680, United States of America; 5 Sociedad Ambiente Marino, University of Puerto Rico, San Juan, Puerto Rico, 00931, United States of America; 6 Universidad del Turabo, Escuela de Ciencias Naturales y Tecnología, Gurabo, Puerto Rico, 00778, United States of America; University of Western Australia, AUSTRALIA

## Abstract

Reef corals typically contain a number of pigments, mostly due to their symbiotic relationship with photosynthetic dinoflagellates. These pigments usually vary in presence and concentration and influence the spectral characteristics of corals. We studied the variations in pigment composition among seven Caribbean shallow-water Scleractinian corals by means of High Performance Liquid Chromatography (HPLC) analysis to further resolve the discrimination of corals. We found a total of 27 different pigments among the coral species, including some alteration products of the main pigments. Additionally, pigments typically found in endolithic algae were also identified. A Principal Components Analysis and a Hierarchical Cluster Analysis showed the separation of coral species based on pigment composition. All the corals were collected under the same physical environmental conditions. This suggests that pigment in the coral’s symbionts might be more genetically-determined than influenced by prevailing physical conditions of the reef. We further investigated the use of remote sensing reflectance (Rrs) as a tool for estimating the total pigment concentration of reef corals. Depending on the coral species, the Rrs and the total symbiont pigment concentration per coral tissue area correlation showed 79.5–98.5% confidence levels demonstrating its use as a non-invasive robust technique to estimate pigment concentration in studies of coral reef biodiversity and health.

## Introduction

Coral reefs are the most biodiverse marine ecosystems of the world. The characteristic variety of colors seen within the living benthic components of a coral reef is mainly due to the array of photosynthetic and photoprotective pigment present in the cells of the flora and fauna, including different types of chlorophylls, carotenoids and xanthophylls. Many of these pigment (or pigment groups) are common among most photosynthetic organisms, including the endosymbiotic dinoflagellates of shallow-water scleractinian reef corals as well as higher plants [1–3].

Due to the similarity of reflected colors and pigment composition, distinction of lower level coral reef benthic taxa (i.e., genus or species) using the spectral signals of photosynthetic organisms has been particularly difficult. As such, past research has concentrated mostly on differentiating between major ecological group levels (i.e., corals, seagrasses, mangroves, etc; [4–6] and between general coral color categories (blue vs. brown) [7]. In corals, their colors are mostly due to their association with endosymbiotic dinoflagellates and to a lesser extent, to other associated endolithic flora [5,8]. In fact, the similarity of colors among hard coral species makes remotely-sensed study of coral reefs particularly challenging as their identification in imagery is based on the differences, if any, of their reflectance spectra.

Despite the availability and generalized use of chemical analysis techniques such as High Performance Liquid Chromatography (HPLC) and spectrophotometry in pigment studies of corals and other reef organisms, usually the results are presented in terms of only the main pigments (such as chlorophyll *a*, chlorophyll *c*_*2*_, peridinin, diadinoxanthin and diatoxanthin) [9–13] and very few attempts have shown the whole spectrum of pigments that may be found within the coral holobiont [8,14–15]. In fact, there is a need for increasing the size of HPLC measured pigment databases for diverse coral species and furthering knowledge of healthy coral pigment concentrations [12]. This is fundamental to the development of techniques that may be used to track changes in reef “health” and biodiversity. Additionally, high genetic diversity in symbionts [16] points to a need to reassess the pigment composition of the different clades. Here, we performed detailed pigment composition analysis of seven Caribbean shallow-water scleractinian coral species based on HPLC and spectrophotometric techniques and use multivariate statistical analysis to test for differences or similarities in pigment groups among the species. Additionally, we propose the use of remote sensing reflectance (Rrs) as a potential non-invasive tool to estimate coral pigment concentration. This may further aid in reef management as a field tool to assess changes in pigment concentrations and indications of reef stress including disease and bleaching.

## Methodology

### Collection Site

Samples were collected from two patch reefs (Enrique [17°57.295 N, 67°03.193 W] and San Cristóbal [17°56.592 N, 67°04.663 W] reefs) within the La Parguera Natural Reserve in Southwestern Puerto Rico after obtaining the appropriate collection permit from the Puerto Rico Department of Natural and Environmental Resources. Both reefs are characterized by a back-reef shallow-water lagoon (0–4 m deep); a reef front dominated by fire corals (*Millepora* sp.) and dead staghorn (*Acropora cervicornis*), elkhorn (*Acropora palmata*) and finger (*Porites* sp.) branches; and a fore-reef zone that extends down to 20 m depth. The back-reef is dominated by a sandy bottom with isolated branched and massive hard coral colonies, gorgonians, and seagrasses. Dead corals on this zone are usually colonized by turf and brown (mainly *Dictyota* sp.) algae with interspersed brunches of the calcareous green alga *Halimeda* sp. The fore-reef zone is dominated by massive species (mainly *Orbicella annularis*, *O*. *faveolata*, *Pseudodiploria strigosa*, *P*. *clivosa*, *Colpophyllia natans*, *Siderastrea siderea*, and *Montastraea cavernosa*). For this study, all the samples were obtained from the back-reef zone of both patch reefs.

### Spectral Analysis

Remotely sensed spectral information was collected *in situ* (between 0930–1100 hrs local time) before coral sampling using a GER-1500 portable field spectroradiometer enclosed within a custom made underwater housing (SpectraVista Corp.). The GER-1500 has a spectral range of 278–1094 nm with 512 spectral bands and a Full Width at Half Maximum (FWHM) equal to 2.8 nm). The housing was held by a diver at a 45° angle and a distance of 2.5 cm away from the target to ensure minimal or no signal contamination due to the presence of dissolved or particulate matter in the water column. Five replicate radiance measurements were obtained from each coral colony and used to study possible differences in reflectance within each sample. Additionally, radiance measurements were obtained from a 50% opaque diffuse barium sulfate (BaSO_4_) reference Spectralon^®^ panel (Labsphere Inc.) immediately after sample spectral data collection at the same distance to correct for any atmospheric or wave lensing effects. Coral-leaving radiance was converted to remote sensing reflectance (Rrs) using: Rrs = Lc/Eg, where L_c_ is the coral-leaving radiance, and Eg = πL_p_/Gc with Lp is the diffuse surface panel-leaving radiance and Gc is the calibration factor of the diffuse panel. The spectra were smoothed with a low pass Savitzky-Golay filter [17–18] to eliminate spectral variability at scales shorter than 4 nm.

### Coral Sampling

**Fig 1:**
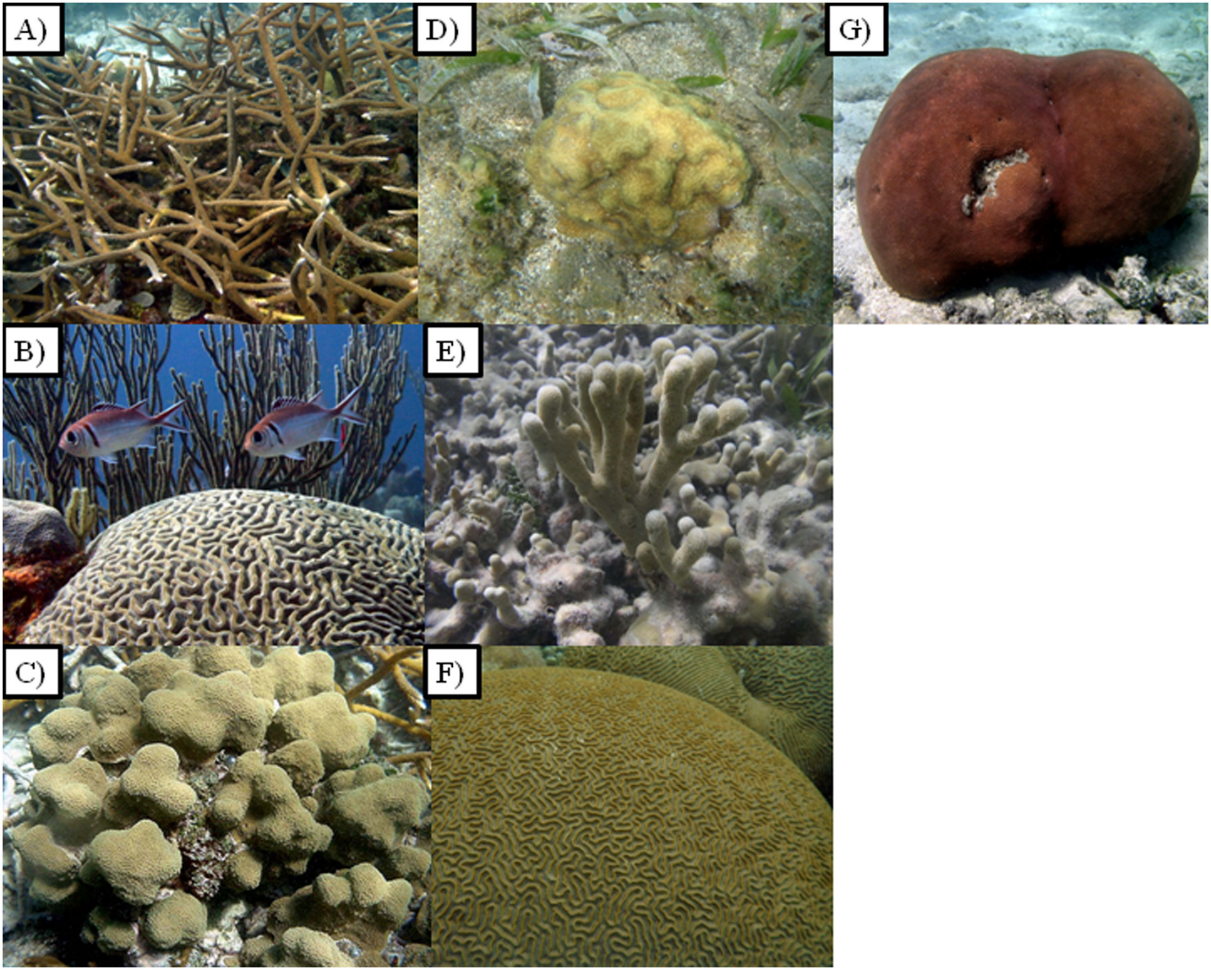
Coral species studied. A) *Acropora cervicornis*; B) *Colpophyllia natans*; C) *Orbicella annularis*; D) *Porites astreoides*; E) *Porites furcata*; F) *Pseudodiploria strigosa*; G) *Siderastrea siderea*.

Samples from seven Caribbean shallow-water coral species (Fig 1) (*Acropora cervicornis*, *Colpophyllia natans*, *Pseudodiploria strigosa*, *Orbicella annularis* (these two species formely known as *Diploria strigosa* and *Montastraea annularis* [19], respectively), *Porites astreoides*, *Porites furcata* and *Siderastrea siderea*) were collected. In the case of the branching corals *A*. *cervicornis* and *P*. *furcata*, one branch was collected from each of seven “visibly healthy” colonies per species at 1 m depth from the back-reef area of San Cristóbal Reef. Colonies sampled were spaced at least 10 m apart to avoid, as much as possible, pseudo-replication or sampling of genetically similar ramets. Here, we define a “visibly healthy” colony as one not showing any signs of disease, discoloration or bleaching [12] compared to the rest of the colony or its neighbors. In the case of massive corals, a 4 cm (diameter) sample was collected with a pneumatic drill attached to a SCUBA tank. Depending on the availability, 4–7 samples were collected from each of the five massive coral species studied. The collection permit from the Puerto Rico Department of Natural and Environmental Resources limited our sampling to ≤ 7 samples per species. The scar left by the drilling was covered with hydraulic cement to avoid colonization of turf algae or any other opportunistic organism. All the samples were transported in aerated seawater in sterilized Whirl-Pak plastic bags and transported to an outdoor aquarium located at the University of Puerto Rico’s Department of Marine Sciences Magueyes Island Field Station (UPR) for immediate processing. The whole transportation process took approximately 30–40 minutes.

Samples were processed for pigment extraction immediately upon arrival at the UPR. In the case of the *A*. *cervicornis* and *P*. *furcata* branches, the white tip of each branch was discarded as it typically contains only a negligible amount of zooxanthellae and pigmentation. All samples (branched and massive) were examined for the presence of any epiphytes which, if detected, were carefully removed from the skeletons with sterilized forceps before any pigment extraction.

Coral tissue and algal cells were removed by airbrush, with the resulting slurry homogenized with a high-speed tissue homogenizer (Biospec Products, Inc.). The solution was separated in two aliquots (5 ml each): one for pigment analysis and the other for symbionts quantification.

### Pigment Analysis

Algal cells were separated from the coral tissue by centrifugation at 5,000 RPM for 10 minutes. Algal pellets were collected and frozen at -80°C until pigment analysis. Algal pellets were resuspended in 100% methanol for pigment extraction, and kept in the dark for 24 hrs at 4°C to prevent photo-oxidation [20]. A second 20-min extraction in the dark at 4°C was performed to extract any remaining pigments. The same procedure was followed with the coral tissue portion to allow for extraction of pigments associated with other endolithic microscopic organisms. Photosynthetic pigments and UV-absorbing compounds were separated by injection through a Sep-Pak C_18_ cartridge [8].

Reverse-phase HPLC was performed to identify the individual pigments within each sample. Pigments were separated in a Shimadzu VP series HPLC system using a gradient system of 80:20 methanol:ammonium acetate (pH 7.2, v/v), 90:10 acetonitrile:water, and 100% ethyl acetate with a Restek^®^ C_18_, 25 cm × 3.9 mm-inner diameter, 5 μm particle size column at a flow rate of 1.0–1.6 ml min^−1^ for 35 min. Eluting peaks were detected using the absorbance spectra at 436 nm for carotenes and chlorophylls following established protocols [21–23]. Peaks were integrated, and quantification of individual pigments was accomplished using peak areas and calibration factors determined by analysis of authentic standards of chlorophyll *a* and lycopene (Sigma Corp.). Individual pigments were identified using published signatures, retention times and their respective peak maxima [20–22, 24–25]. Coral tissue area was used for normalization of pigment concentrations and determined following the aluminum foil technique of [26]. Concentrations of pigments were expressed in μg·cm^−2^.

### Symbiont Quantification

Symbiont cells were fixed in 10% formalin in seawater solution for 24 hrs and then rinsed for another 24 hrs in deionized water. To avoid any effects of diel pattern cell divisions [27], all the samples coming from any single coral species were fixed at the same time of day. To further separate the symbiont cells from the coral tissue, the samples were re-homogenized at 7,000 RPM. Afterwards, the slurry was decanted into a 50-ml centrifuge tube with 5 ml of DIW, centrifuged at 5,000 RPM and the supernatant was discarded. The remaining pellet containing the symbiont cells was re-suspended in 2 ml of filtered seawater. The cells were counted in triplicate in a Reichert hemocytometer and averaged. Symbiont concentration was expressed as cells per cm^-2^ of coral tissue.

As we were unable to perform genetic identification of the different symbiont clades found in the studied species, we base our Principal Components Analysis, Hierarchical Cluster Analysis, and further discussion on the most common symbiont clades found in the Caribbean (including Puerto Rico) within colonies living under similar environmental conditions as the ones we sampled here (Table 1).

**Table 1:** Summary of symbiont clades found in the literature for the seven coral species sampled during the present study.

Coral species	Symbiont clade	Depth (m)	Site	References
*A*. *cervicornis*	A3	4	Bahamas	[3]
	A*	4.3–12.7	Multiple **	[28]
	A3	4	Mexico	[29]
	A3	3	Bahamas	[29]
	C12	12	Bahamas	[29]
*C*. *natans*	C1, B6	4	Mexico	[29]
	B6, B9	12	Bahamas	[29]
*O*. *annularis* ^*#*^	B1	4	Bahamas	[3]
	B1, C3	4	Bahamas	[29]
	B1, D1a	14	Bahamas	[29]
	B1, C3, C7	8	Belize	[30]
*P*. *astreoides*	A4a, A3, B1	2.5	Mexico	[29]
	A4a	4	Bahamas	[29]
	A*	4–6	Bermuda	[15]
	A4	2	Belize	[30]
	A4a	8	Belize	[30]
*P*. *furcata*	A4, B1	1.5	Mexico	[29]
	C4	5	Mexico	[29]
*P*. *strigosa* ^*##*^	B1, C1	2.5	Mexico	[29]
*S*. *siderea*	C1	2.5	Mexico	[29]
	C3	2	Belize	[30]

* = Intragenomic specificity not mentioned in the reference.

** = Florida Keys, Puerto Rico, Bahamas, Honduras, Navassa and St. Thomas.

^#^ = identified as *Montastraea annularis* in the mentioned references.

^##^ = identified as *Diploria strigosa* in the mentioned reference.

### Relationship between Remote Sensing Reflectance and Pigment Concentration

We performed regression analyses to study the relationship between remote sensing reflectance (Rrs) of each coral species and both, the total pigment concentration (sum of all pigments, symbiont or non-symbiont origin) and the symbiont total pigment. The area under each reflectance curve was integrated using:

$$\sum_{\lambda =400}^{\lambda =700}0.5\left(\frac{Rrs\lambda 1+Rrs\lambda 2}{\Delta \left(\lambda 2-\lambda 1\right)}\right)$$ (1)

where Rrsλ1, …, Rrsλn is the coral’s Rrs at λ1, λ2…, λn. Total pigment concentration was obtained from the HPLC analysis described above.

### Statistical Analysis

The data for chlorophylls, carotenes and xanthophylls concentration were tested for normality (Kolmogorov-Smirnov test) prior to the statistical analysis. A non-parametric Kruskal-Wallis test was used to test for differences in total pigments, chlorophylls, carotenes and xanthophylls concentration among the seven coral species when the data did not follow a normal distribution. Otherwise, a One-way ANOVA was used for normally distributed data. A Tukey test with pairwise comparisons was used to identify specific differences in pigment concentration among species [31]. A Chi-square test was used to test for differences among the Rrs curves of the seven corals species studied.

Additional multivariate analysis was performed using the PRIMER 6 statistical package (PRIMER-E Ltd.). Principal components analysis (PCA) was performed on the main pigment data to test for variations among species main pigment concentrations (*i*.*e*., Chl *a*, Chl *c*_2_, Per) and pigment groups (chlorophylls, carotenes and xanthophylls). PCA has been used in the past as a data reduction technique and as a means to identify different modes of data [32]. The PCA was chosen as it preserves the total variance while minimizing the mean square approximate errors and it is also used as a means to identify dominant modes of data [33]. PCA transforms the original dataset into a smaller set of uncorrelated variables that represent most of the information in the original dataset. The first principal component accounts for the maximum proportion of the variance of the original dataset, and subsequent components account for the maximum proportion of the remaining variance.

A Hierarchical Cluster Analysis (HCA) was used to test for similarity (or dissimilarity) in pigment concentration among the seven coral species studied. The data matrix consisted of columns representing the concentration and percentage of the main pigment groups, similar to PCA, and the rows representing the seven coral species.

## Results and Discussion

### Pigment Composition Analysis

In total, 27 different pigments were identified in the seven coral species studied (Table 2; S1 Table) (see pigment names abbreviations in Table 2). Of these, peridinin (Per) is the only pigment specific to dinoflagellates [25]. Seven of the 27 pigments were common to all seven coral species (Chl *a*, Chl *c*_*2*_, Per, DcI, DcII, Dd, and P-457). Typically, Chl *c* (*c*_*1*_ + *c*_*2*_) and peridinin are the most abundant accessory pigments in dinoflagellates [34].

**Table 2:** Concentration and average percent of individual pigments per coral colony sampled. Concentrations are expressed as μg cm^-2^ ±1 SD and n = 4–7, depending on the species. Absorption peaks may vary from those found in the literature as these depend on the extraction solvent and HPLC setup. Pigments are listed in alphabetical order. Pigments common to all 7 coral species studied are tagged with an asterisk (*).

Pigment	*A cervicornis*		*C natans*		*O annularis*		*P astreoides*		*P furcata*		*P strigosa*		*S siderea*		Abs Peaks
	[Pigm]	Avg %	[Pigm]	Avg %	[Pigm]	Avg %	[Pigm]	Avg %	[Pigm]	Avg %	[Pigm]	Avg %	[Pigm]	Avg %	
19-hf					2.36±1.2	2.0									446,470
9-cis-Nc					2.64±0.0*	1.4	1.49±0.0*	1.6	1.70±1.2	1.5	2.65±1.4	1.5	2.66±0.9	1.6	422,450
9-cis-neo			2.66±0.0*	1.2							3.71±0.4	1.4			438,467
β,β-car	0.21±0.1	0.5	2.48±0.0*	1.1	0.98±0.5	0.7	2.45±0.4	1.4	0.57±0.4	0.5			0.88±0.1	0.4	449,475
β,ε-car					0.86±0.0*	0.5									446,474
*Chl *a*	13.44±6.3	65.9	65.06±3.7	30.1	44.29±2.0	53.9	78.93±5.7	53.7	30.43±1.5	48.6	60.32±4.3	29.1	28.19±1.9	46.9	432,665
Chl *a* allo	0.24±0.02	0.5	0.73±0.1	0.3	3.65±1.4	4.2			0.52±0.4	0.8	0.53±0.1	1.6	4.94±0.0*	1.3	432,665
Chl *a* epi	1.47±0.6	3.0			2.13±1.1	1.6	1.39±0.0*	0.6	0.10±0.07	0.1			4.82±0.0*	1.3	432,665
Chl *b*					4.36±2.13	3.8	2.40±2.1	1.4					2.02±1.1	1.7	470,652
*Chl *c*_*2*_	2.93±1.1	6.4	5.10±1.2	2.5	8.70±3.7	6.1	11.97±5.7	9.2	12.14±5.9	11.6	20.77±8.6	11.6	18.04±7.9	9.1	452,635
*Dc I	1.17±1.0	1.5	14.12±4.5	4.5	4.2±0.3	3.1	2.40±0.2	1.7	1.97±0.2	1.7	7.54±2.8	3.1	5.50±2.0	2.3	430,457
*Dc II	0.73±0.1	2.0	10.91±0.0*	6.9	4.93±0.4	3.6	3.49±2.0	2.6	3.22±2.1	3.2	5.75±1.8	3.7	7.69±1.4	4.2	430,457
*Dd	1.90±0.8	6.5	2.76±1.1	1.3	2.60±0.3	2.5	0.87±0.1	0.6	0.39±0.1	0.5	0.45±0.0*	0.3	0.91±0.6	0.5	445,476
Dt							3.17±2.38	1.9	1.2±0.8	1.1			1.18±0.3	0.4	452,478
Dn	0.66±0.1	1.0	4.5±2.8	2.1	0.57±0.2	0.6	0.92±0.0*	0.4							438,467
Fc	1.18±0.0*	3.5			2.16±0.6	1.6	1.35±0.0*	0.6	0.76±0.0*	0.7	2.71±2.6	1.6	3.75±2.0	1.6	446,475
Gy	0.45±0.0*	1.9													442,470
Lu	0.34±0.0*	1.5													443,470
Mg-DVP	0.60±0.3	0.6			0.13±0.0*	0.2					1.60±0.0*	0.6			437,624
MV-Chl *c*_*3*_	0.38±0.1	1.4									1.64±0.0*	1.0			447,626
Nc			14.13±3.2	6.8	3.09±2.0	2.3	1.29±0.6	1.0	0.86±0.5	1.0	6.18±3.0	3.1	5.79±2.7	2.4	424,451
*P-457	0.66±0.1	1.9	26.20±8.9	12.4	6.61±3.7	4.8	8.43±4.2	6.2	5.50±0.5	6.1	17.50±7.3	9.6	19.64±1.6	6.8	457
P-468							1.55±0.0*	1.7			2.78±0.0*	1.7			468
*Per	5.01±0.4	7.0	79.42±1.9	30.8	26.06±1.2	18.2	28.28±1.8	20.4	20.28±1.1	23.6	69.44±2.5	36.3	68.95±4.4	24.9	475
Sipho			1.41±0.0*	0.6			0.96±0.0*	1.6	0.59±0.2	0.6	3.72±3.1	1.6			441,461
Vio					1.4±0.0*	1.7	0.43±0.2	0.3	0.37±0.1	0.4					440,470
Zea	1.17±0.0*	2.6	4.76±0.0*	2.2	2.59±0.0*	3.3	1.29±1.1	1.1					4.80±1.5	1.3	449,475

Abbreviations: 19-hf—19-hexanoloxyfucoxanthin; 9-cis-Nc—9-cis-Neochrome; 9-cis-neo— 9-cis-Neoxanthin; β,β-car—β,β carotene; β,ε-car—β,ε carotene; Chl *a*—Chlorophyll *a*; Chl *a* allo—Chlorophyll *a* allomer; Chl *a* epi—Chlorophyll *a* epimer; Chl *b*—Chlorophyll *b*; Chl *c*_*2*_—Chlorophyll *c*_*2*_; Dc I and II—Diadinochrome I and II; Dd—Diadinoxanthin; Dt—Diatoxanthin; Dn—Dinoxanthin; Fc—Fucoxanthin; Gy—Gyroxanthin dodecanoate ethanoate; Lu—Lutein; Mg-DVP—Magnesium 2,4-divinyl pheoporphyrin a monomethyl ester; MV-Chl *c*_*3*_ —Monovinyl Chlorophyll *c*_*3*_; Nc—Neochrome; Per—Peridinin; Sipho—Siphonaxanthin; Vio—Violaxanthin; Zea—Zeaxanthin.

The occurrence of pigments such as Zea, Vio, and Lu, among others is indicative of the contribution of other types of photosynthetic organisms (such as microscopic endolithic algae) present in the samples, despite the careful visual examination of each sample during the pigment extraction protocol. Such pigments can be found in several algal groups [24]. On average, these pigments contributed 8–13% of the total pigment composition, depending on the species, with branched species (*A*. *cervicornis* and *P*. *furcata*) containing the smaller fraction of pigments associated with other photosynthetic organisms.

Here, we expand on previous studies on Caribbean shallow-water coral pigment characterization [8,14]. Our group previously demonstrated significant differences in pigment composition among *Acropora cervicornis* and *Porites porites* collected under similar conditions in Puerto Rico [8]. The present study reveals similarly robust differences among additional coral species. Interestingly, while the pigments associated with the presence of additional photosynthetic organisms in the samples contributed up to only 13%, the variety of such pigments found here shows that the coral holobiont may be composed of an array of different organisms and not only corals and their endosymbiotic dinoflagellates. Additional evidence is the presence of pigments associated with the energy-dissipating xanthophyll cycle which in dinoflagellates is composed of Dd and Dt, whereas in other algae and higher plants is composed of Zea, Viol and antheraxanthin (the latter was not found in any of the species during the present study). We found most of these accessory pigments in the present study. Other pigments such as Fu and Lu are most likely indicative of the presence of Pheophytes and Chlorophytes, respectively [35–36]. Fucoxanthin is also usually confined to diatoms, Prymnesiophytes, Raphidophytes and Chrysophytes and 19’-hexanoyloxyfucoxanthin is the major carotenoid of some coccolithophores although these two pigments have also been identified in dinoflagellates that contain endosymbionts themselves [24–25,37]. The presence of chl *b* in some samples indicates presence of endolithic algae, particularly Chlorophytes [25]. Earlier studies have also reported the presence of chl *b* in coral samples. For example, Apprill et al [12] detected chl *b* in samples of *Porites lobata* and *P*. *lutea* from Hawaii and estimated its contribution to the total pigment pool to be around 1%. In our study, chl *b* was detected in samples from three species (*O*. *annularis*, *P*. *astreoides* and *S*. *siderea*) and its contribution ranged from 1.4–3.8% to the total pigment pool of particular colonies.

Pigments such as chl *a*, chl *c*_*2*_ and peridinin serve to capture light whereas others like Dd, Dt, Dn and β,β-carotene are part of the photoprotective mechanism of photosynthetic organisms [38–40]. Xanthophyll cycling causes Dt and Dd to fluctuate with changes in light conditions [41]. Under high light, Dd is de-epoxidated to Dt, which protects PSII from excess excitation and the consequent oxidative damage, while in low light Dt is transformed back to Dd [15]. Additionally, the degree of xanthophyll cycling is quite different between *Montastraea (Orbicella) annularis* with type B1 symbiont and *Acropora cervicornis* hosting type A3 at the same depth [3]. In contrast, Hennige et al [34] studying the photoacclimation characteristics of several *Symbiodinium* types including type A3 and B1 under two different photon flux densities found little variation in the relative abundance of pigments among the symbiont types studied, with only a significant difference in the de-epoxidation state (Dt/Dd + Dt). Our results support the findings of Warner and Berry-Lowe [3] as even though we did not find Dt in either species, the concentration (and percentage) of Dd differed between both species. The time of collection might have influenced these results as the proportions of Dt and Dd usually vary through the day depending on the state of non-photochemical quenching within the photosynthetic apparatus of the symbiotic dinoflagellates. Failure to detect Dt in both species might have also been caused by a rapid conversion to Dd during the time from sample collection to processing.

Based on our previous experiences, we decided to use a two-phase extraction process (24 hrs at first and then for 20 minutes the day after to extract any remaining pigments), as shown in the methodology. This ensured the extraction of basically all the pigments but also may have incorporated some artifacts. For example, the presence of allomers and epimers of chl *a* may have been a consequence of this long extraction as these usually do not appear in short-term extractions [37]. For instance, McDougall et al [42] identified chl *a* allomers and epimers as possible biomarkers of stress (or at least as part of the photo-adaptation process of the zooxanthellae) in *Goniastrea aspera*. Similarly, even though we processed all the samples as soon as they were brought to the laboratory, some alteration products of accessory pigments, such as Dc I and II, were present in almost all samples of all species, probably as a consequence of handling stress, change in the light regime for a short period of time, and alteration of the photosynthetic apparatus, among other factors.

**Fig 2:**
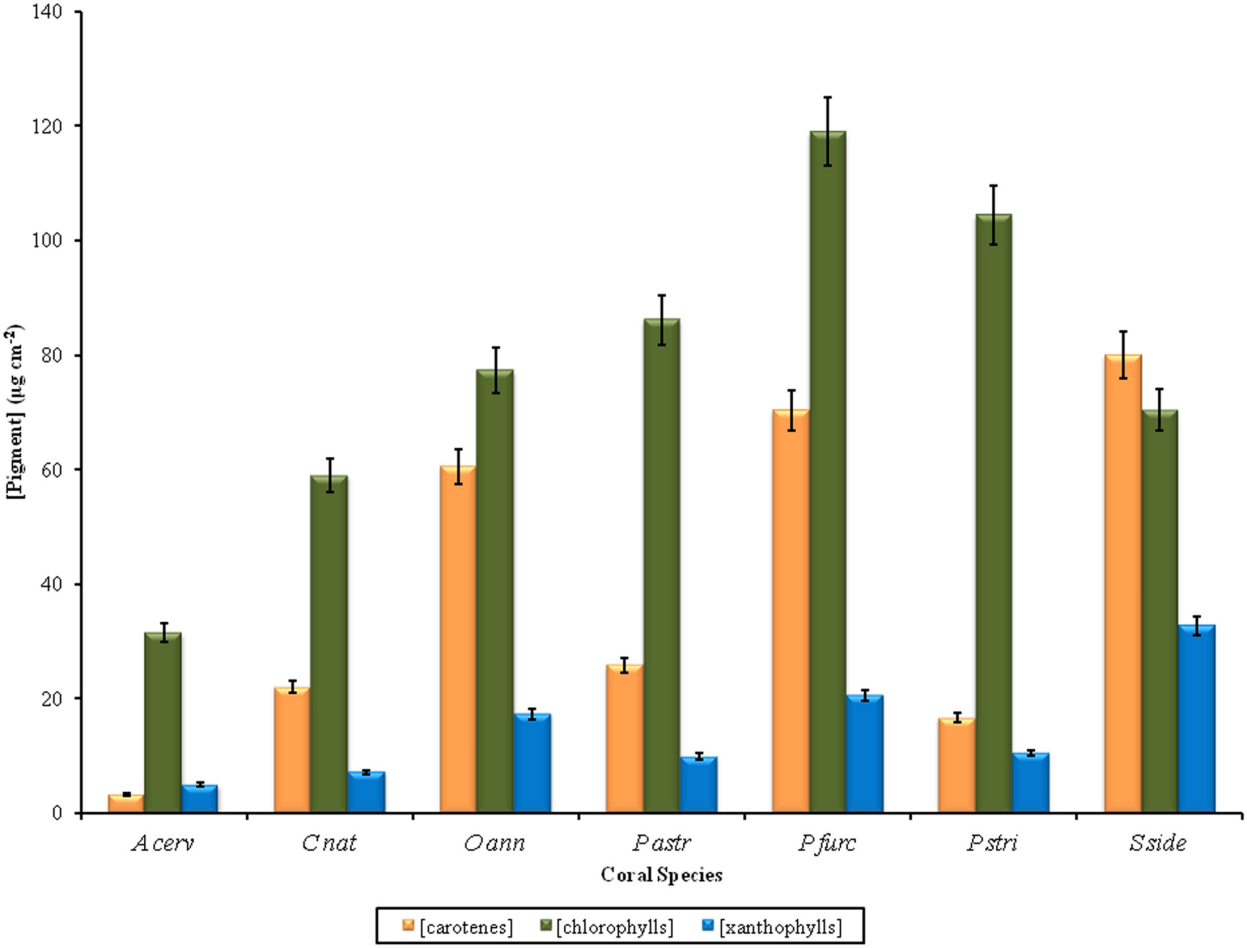
Pigment contents among the seven coral species studied. Variability in concentration of pigment groups among species. Coral species abbreviations: *A cerv—Acropora cervicornis; C nat—Colpophyllia natans; O ann—Orbicella annularis; P astr—Porites astreoides; P furc—Porites furcata; P stri—Pseudodiploria strigosa; S side—Siderastrea siderea*. Error bars indicate ±1SD.

When considering the contribution of the endosymbiotic dinoflagellates to the coral pigment complexes, chlorophylls were the dominant pigment group (both in percentage and concentration) in all the corals with the exception of *Siderastrea siderea*, where carotenes dominated (particularly peridinin) (Fig 2). There were significant differences between species in terms of chlorophylls concentration (One-Way ANOVA, F = 5.04, p = 0.001) and percentage (Kruskal-Wallis, H = 27.24, p<0.0001), carotenes concentration (One-Way ANOVA, F = 10.43, p<0.0001) and percentage (Kruskal-Wallis, H = 30.49, p<0.0001), and xanthophylls concentration (One-Way ANOVA, F = 6.22, p<0.0001) and percentage (Kruskal-Wallis, H = 13.53, p = 0.035). This is consistent with past findings where the variability among pigment percentages was smaller than that of their respective concentrations within colonies of the same species living at similar depths [12]. Additionally, our results coincide with those of Myers et al [10] who found that “healthy” colonies of *A*. *cervicornis* had the lowest relative total pigment, chl *a* and peridinin density among the coral species studied which included *M*. *(Orbicella) annularis* and *P*. *astreoides*.

**Fig 3:**
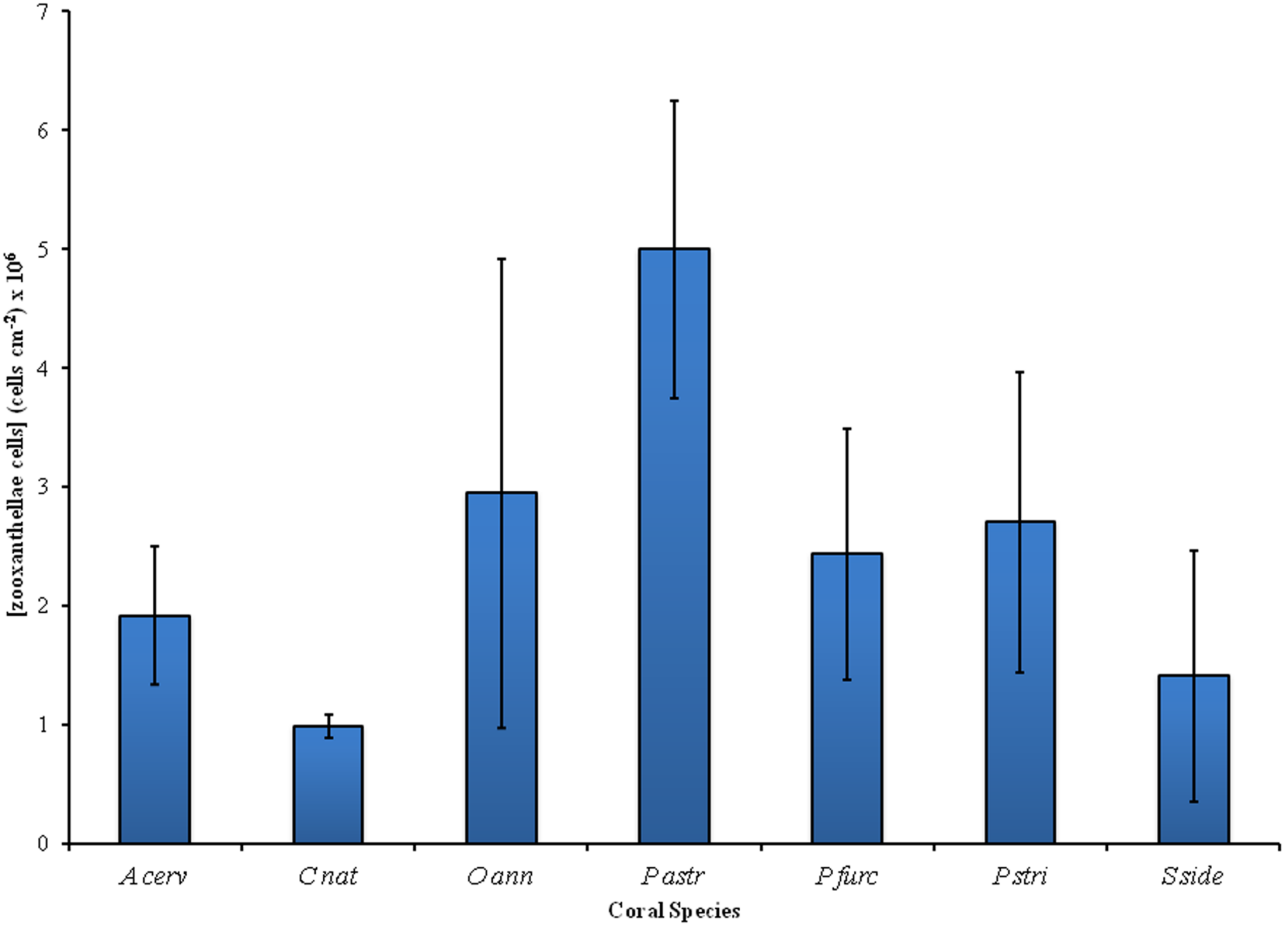
Concentration of symbiont cells per coral tissue area per species. Coral species abbreviations: *A cerv—Acropora cervicornis; C nat—Colpophyllia natans; O ann—Orbicella annularis; P astr—Porites astreoides; P furc—Porites furcata; P stri—Pseudodiploria strigosa; S side—Siderastrea siderea*. Error bars indicate ±1SD.

The average concentration of symbiont cells within the coral tissues ranged from 985,705 to 5,001,700 cells per cm^-2^ of coral tissue and also differed significantly between species (Fig 3; One-Way ANOVA, F = 7.40, p<0.0001) with *P*. *astreoides* containing the highest average concentration of symbionts and *C*. *natans* the lowest. The overall high variability in symbiont concentration found within most studied species is reflective of the small sample size. Nonetheless, keeping in mind that all samples were collected at the same depth (1m), the range in symbiont concentration among species may reflect the influence of different skeletal arrangements and the consequent differences in light regime reaching the endosymbiotic dinoflagellates within the coral tissue [43]. This in turn affects the concentration and distribution of symbiont cells within the coral tissues and their respective photosysnthetic pigment array. For instance, a coral such as *C*. *natans* has a particularly thin meandriod skeletal structure with large polyps while *P*. *astreoides* has a denser smoother skeleton with small polyps. This is reflected in their differences in symbiont cocentration with the latter containing 5X that of the former.

### Principal Component Analysis (PCA) and Hierarchical Cluster Analysis (HCA)

**Fig 4:**
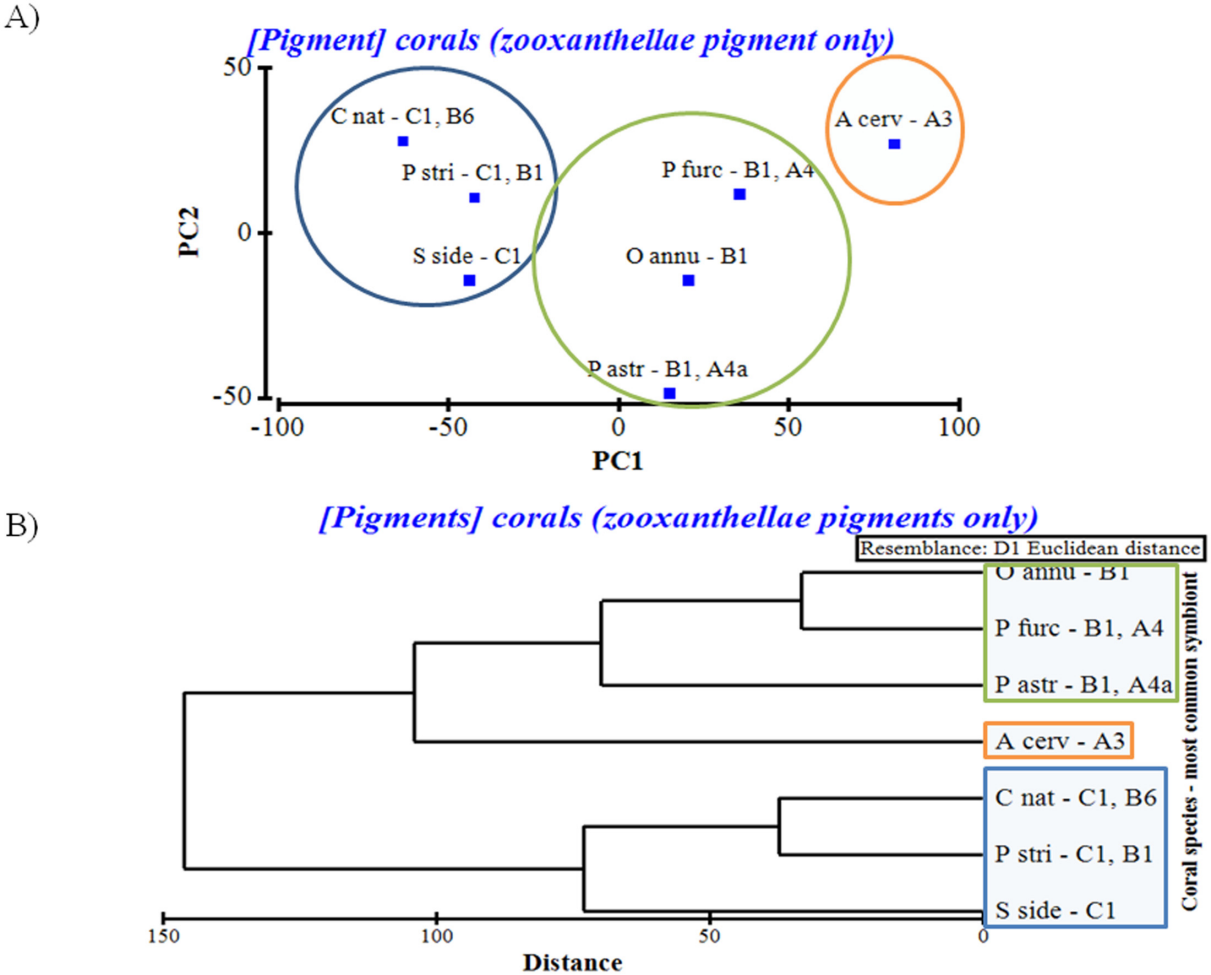
Multivariate statistical analysis of coral pigments. A) PCA and B) HCA showing the grouping of the seven coral species based on the concentration of the main photosynthetic pigments (chlorophylls, carotenes and xanthophylls). The most common symbiont clade found in each coral species (based on published literature) is shown next to the coral species name.

The PCA resulted in a matrix of the main individual pigment concentrations, relative individual pigment composition, pigments groups and relative pigment group composition. We evaluated 12 principal components (PC) for symbiont pigment: total concentration of chlorophylls, carotenes and xanthophylls; percentage of chlorophylls, carotenes and xanthophylls; concentration and percentage of the three main pigments (chlorophyll *a*, chlorophyll *c*_*2*_ and peridinin). These principal components were evaluated against the PC eigenvectors, which showed which of these PC were the most deterministic in grouping of the seven coral species studied. The first two PC explained 87.2% of the variation between coral species. PC1 (a combination of the concentration of peridinin and the percent of chlorophylls) explained 68% of the variation, while the PC2 (a combination of the concentration of chlorophylls and the concentration of carotenes) explained 19.2% of the variation (Fig 4A). The HCA showed that the pigment composition of Caribbean shallow-water reef corals is directly related to the type of symbiont clade that they harbor (Fig 4B). In fact, the analysis indicates that while photosynthetic pigment concentration in a particular coral colony may be influenced by the light conditions [3,44–45], the relative proportion of the main groups is directly related to the symbiont clade present within the coral tissue, and as such, this can be an alternate way of separating coral and symbiont taxa. Both the PCA and HCA confirmed the same distinct separation of the seven coral species based on the concentration of the main photosynthetic pigments. Our findings are based on reported symbiont clades for the studied species. A genetic analysis may further confirm these findings.

There are 9 sub-generic *Symbiodinium* clades [46], with over 400 distinct internal-transcribed spacer-2 (ITS-2) rDNA “types” described so far [29–30,47]. Yet, there seems to exist a prominent ecological zonation in several of the most abundant clades, and it is conceivable that relative pigment concentrations drive this zonation [29]. In many coral species, sea surface temperature and irradiance have significant effects on algal density and pigment concentration of reef corals [15,44]. For instance, *Symbiodinium* clade B1 is considered a “generalist” symbiont on Caribbean reefs residing in several host taxa and across a wide light/depth range, while clade A tends to prefer shallower locations. This is consistent with the fact that *Acropora cervicornis* (which typically hosts clade A3) tends to live in shallow waters [28] although it is also seldom found up to about 20 m of depth [45]. Yet, under high temperature and sediment zones, *A*. *cervicornis* harbors clade D [28]. The prevalence of clade B among hosts and depth ranges might be due, among other factors, to the small cell size (it is morphologically the smallest *Symbiodinium* group) [48–49] and its photosynthetic plasticity. As shown in Table 1 above, for most of the coral species studied here, they seem to harbor several clades (but see [50] on the prevalence of coral species fidelity to a narrow subset of a single zooxanthellae clade worldwide). We kept our samples collection to a specific narrow depth range (around 1m), hence, here we did not accounted for depth effects on symbiont population among coral colonies. Additionally, there is little information on how the symbiont population among the studied species vary along the Puerto Rico shelf [28] and the range of environmental factors (e.g., depth range, light transparency, distance to shore) occurring there. Hence, such study can bring light on the biogeography of symbiont populations at a local (e.g., within an island shelf) level as oppose to the region (e.g., Caribbean) or worldwide (e.g., tropics).

Past estimates of coral-algal specificity obtained using conventional molecular methods showed that probably less than 25% of all coral species associate with multiple *Symbiodinium* clades [29] and this seems to vary depending on the geographical basin where the coral species reside (i.e., Caribbean vs. Indo-Pacific) [29,51]. More recent data obtained with real-time polymerase chain reaction (RT-PCR) analyses have shown that on average, and apparently independently of the geographic region, 68% of the coral species host at least two symbiont clades at any given time [47]. From the species sampled in the present study, Silverstein et al [47] found that *Acropora cervicornis* and *Colpophyllia natans* may host 2–3 and 2–4 symbionts at a time, respectively. We were not able to run a genetic analysis of the symbionts clades during the present study, hence, we cannot deny nor confirm the presence of multiple clades within our samples. Additionally, we were only allowed to collect a limited number of samples per coral species and this can also affect our overall estimations of relative pigment concentrations/percentages and its estimation with remote sensing reflectance (see below).

### Remote Sensing Reflectance

**Fig 5:**
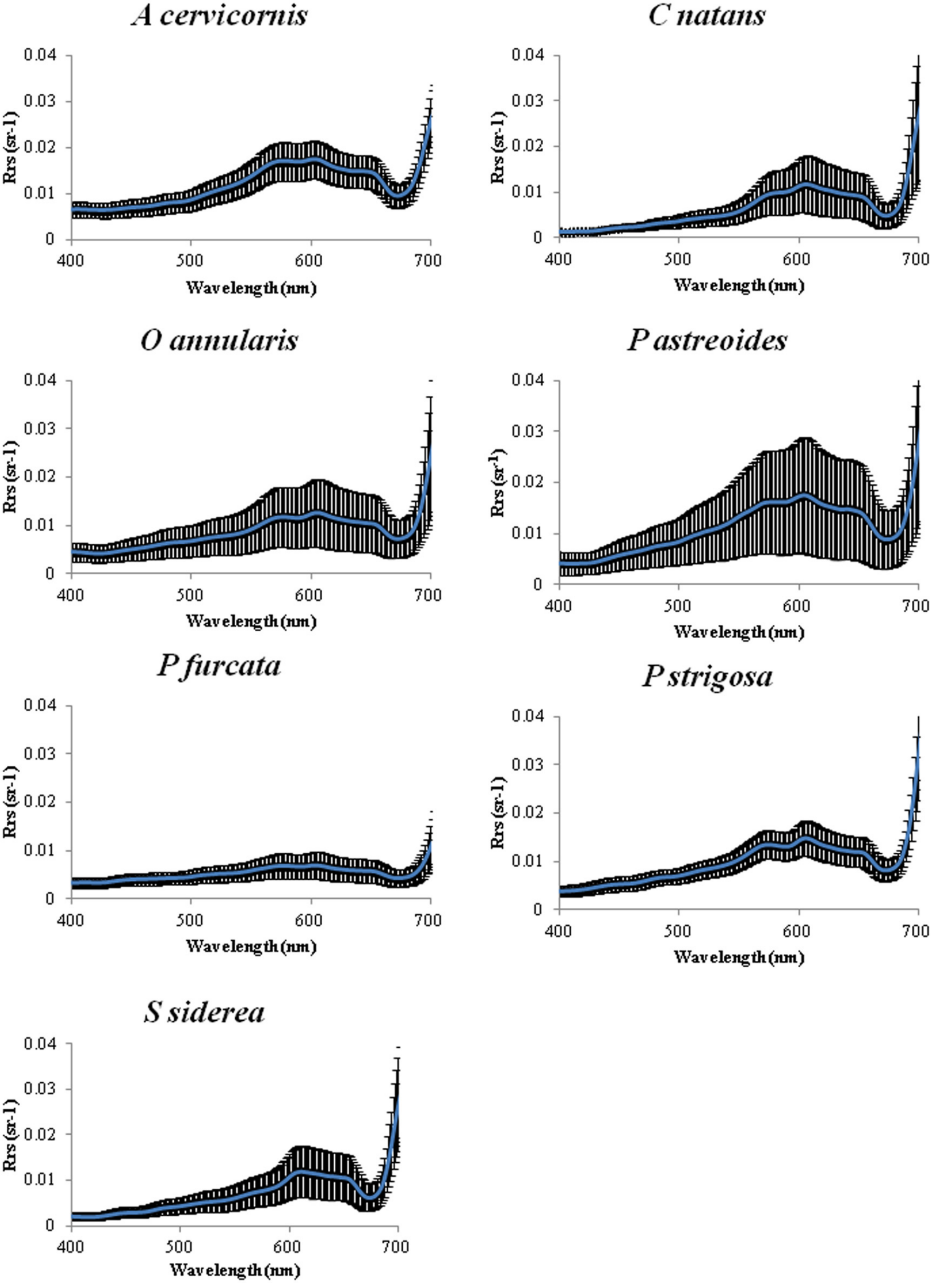
Remote sensing reflectance for all seven species. The blue line represents the average Rrs with ±1SD shown as the black shadow. n = 5 for each species.

While there were differences in magnitude, all coral species showed the typical reflectance peaks around 572, 604 and 644 nm (Fig 5). Nonetheless, a Chi-square test showed a statistically significant difference among the Rrs curves of all seven coral species (χ^2^ = 23224, p<0.0001). Our past study showed correlations of some of the reflectance peaks (after a derivative analysis) with the presence of certain pigments [8]. Whether the Rrs is more influences by symbionts or pigment concentration seems to be species-specific. This is evidenced, for instance, in our *C*. *natans* and *P*. *astreoides* samples as whereas both showed the highest Rrs within the visible range (Fig 5), they differ significantly in symbiont and chlorophylls concentration. Contrarily, while *P*. *furcata* and *P*. *strigosa* contain similar symbiont and chlorophylls concentration, the Rrs of *P*. *furcata* is significantly lower than that of *P*. *strigosa*. This may be the result of a combination of different factors. First, the differences in skeletal density and structure mentioned above affects the distribution of symbionts and their respective pigment. Additionally, colony form (branched vs. massive) may lead to an internal “package effect” (overlap among the optical cross-sections of pigments within cells and mutual shading) [52], which affects the internal light regime reaching the symbionts and, hence, the absorption of photons. Such might be the case of *P*. *furcata* and *C*. *natans* as these showed the lowest correlations between the Rrs area and pigment concentration.

**Fig 6:**
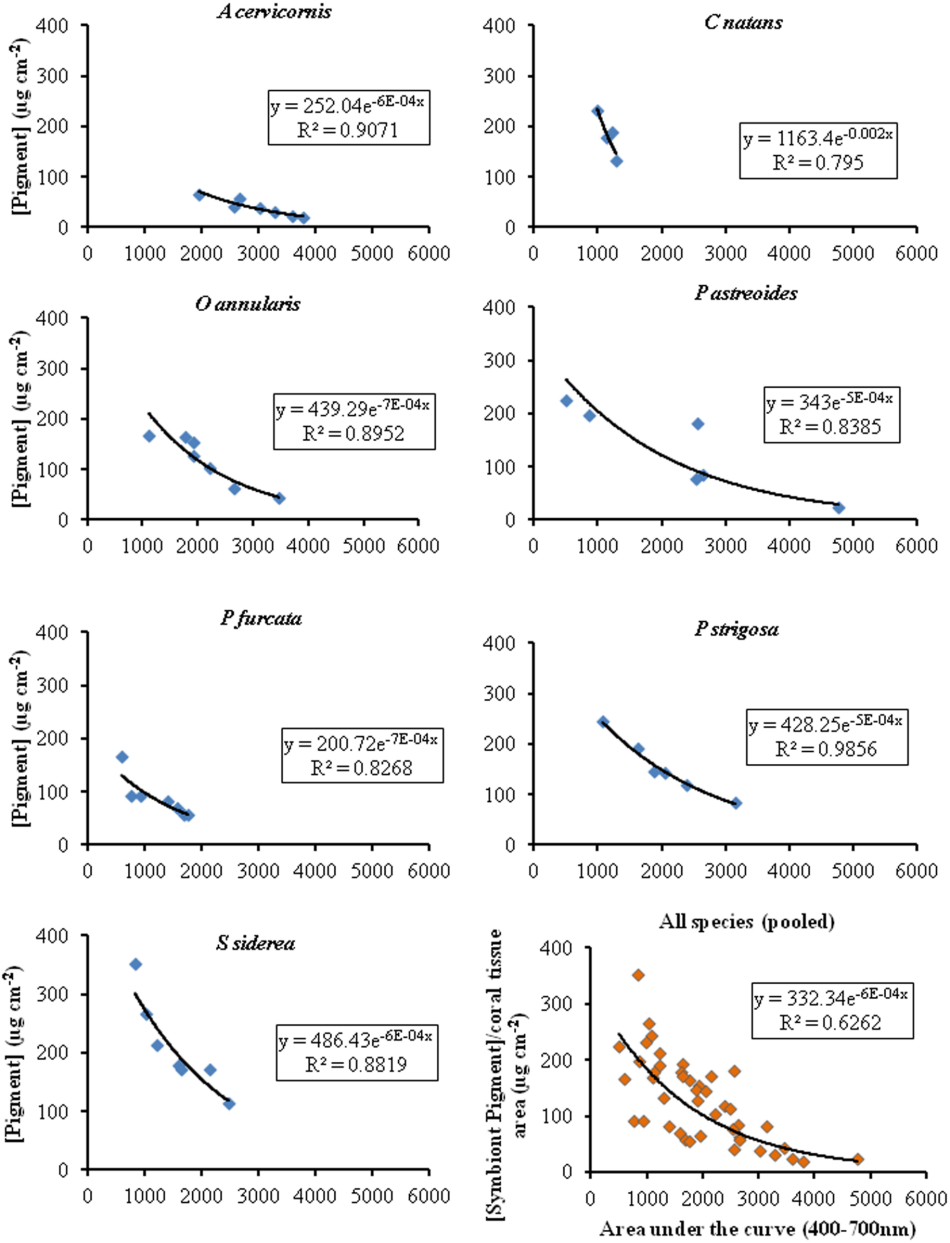
Relationship between the area under the reflectance curve and total symbiont pigment concentration. The x-axis represents the integration of the reflectance curve from 400–700 nm. The lower right graph shows the pooled data from all seven species.

Hochberg et al [5] grouped all the corals measured from the Pacific and the Caribbean into two basic shapes of spectral reflectance, “brown” and “blue” modes and postulated that “brown” mode reflectances are more determined by the symbiont’s pigment absorption whereas “blue” mode reflectances are more influenced by the expression of non-fluorescent coral-host pigment. Among the species exhibiting “brown” modes were: *Montastraea (Orbicella) annularis*, *Diploria* (*Pseudodiploria) strigosa*, *Porites astreoides*, and *Siderastrea siderea*, although the latter also exhibited “blue” modes. Based on our results, the spectra of *Siderastrea siderea* shown in Fig 6 accommodates to the “blue” mode described by [5] for this species. Host pigmentation can also generate spectral variations in the visible wavelengths [53], for example green fluorescent proteins (GFP) and other GFP-like pigments absorb and fluoresce in shorter and longer wavelengths, respectively, potentially contributing to the reflectance spectra of corals [54]. The contribution of fluorescent pigments to the apparent reflectance in corals is a factor which may be significant but has so far only been partially addressed [55–56]. The function of pigments within the coral tissue (i.e., GFP, GFP-like, pocilloporins, and others) is still under debate with photoprotective, energy transfer and chemical defense roles being proposed [13,49,57–61].

The colonies of *S*. *siderea* studied here were among those containing the least number of symbiont cells per coral tissue area (Fig 3) and, based on the findings of Hochberg et al [5] and Mazel and Fuchs [56], having a “blue” mode reflectance may be a consequence of this low symbiont concentration and possibly a higher influence of the presence of GFP or GFP-like host pigments present within the tissues. Nonetheless, we have no evidence from the HPLC analysis to support this as the present study was not intended to account for the presence of these host pigments.

### Rrs as a Potential Estimator of Coral Pigment Concentration

**Fig 7:**
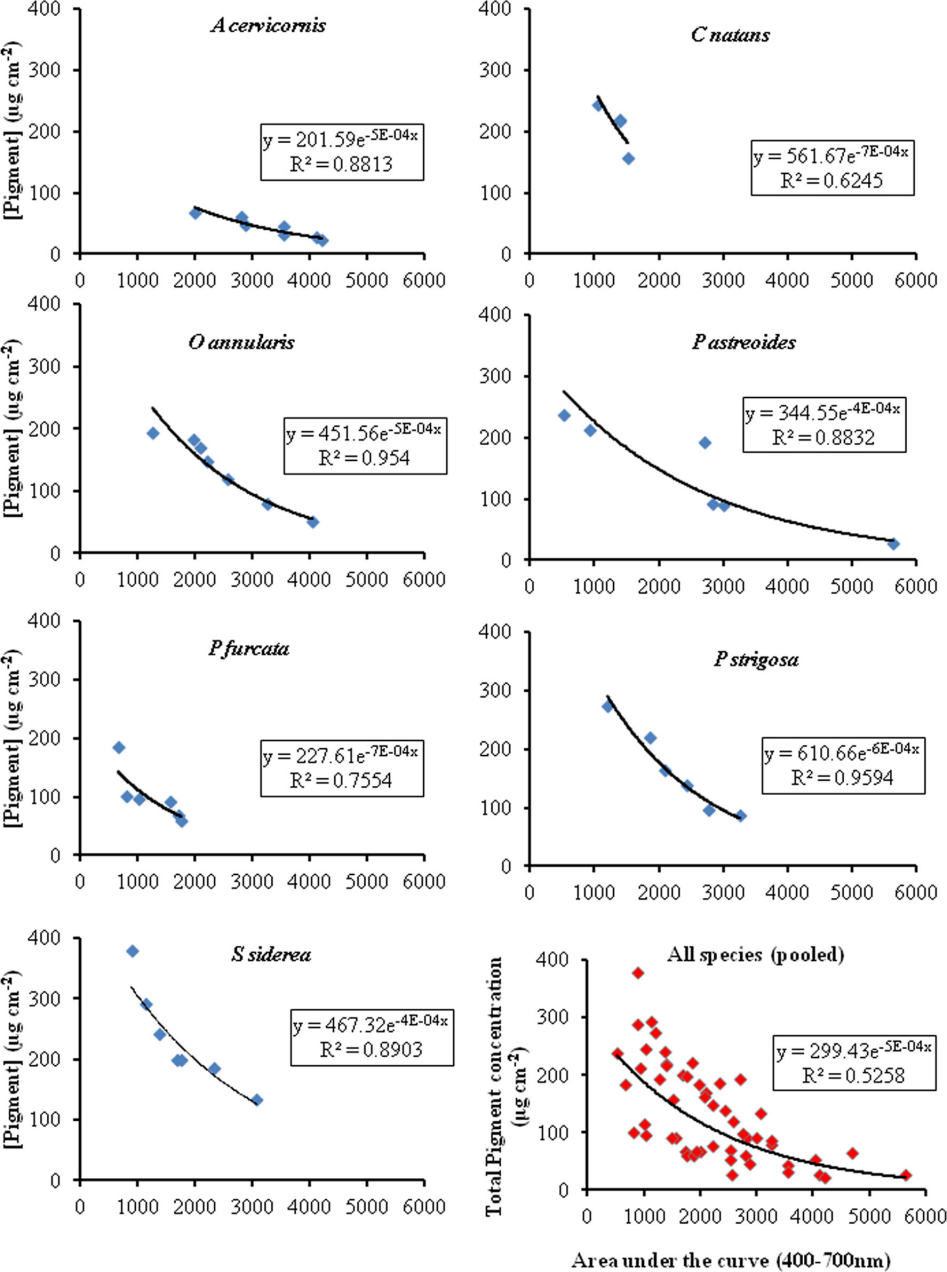
Relationship between the area under the reflectance curve and total pigment concentration (symbiont + other contributors). The x-axis represents the integration of the reflectance curve from 400–700 nm. The lower right graph shows the pooled data from all seven species.

Our previous work with *Acropora cervicornis* and *Porites porites* showed the potential use of reflectance to estimate pigment. Here, we applied a similar technique, but using Rrs instead, to a larger number of species. An integration of the area under the Rrs curve was used to evaluate its use within the visible range (400–700 nm) as a proxy for estimating total pigment concentration in reef corals. Fig 6 shows that based on the total symbiont pigments composition and depending on the species, we found 79.5–98.5% predictability with an overall prediction effectiveness of 62% when all the species data is pooled. The prediction effectiveness diminishes when the pigments from additional sources are added (62–95% with an overall of 52%) (Fig 7). While we were limited in the number of colonies that we could sample, a higher sample size (hence, potentially more variability in symbiont and pigment concentration), either from the same sites or from additional sites, may produce different results. Nevertheless, our data shows a persistent pattern exemplified by the high Rrs area and pigment concentration correlations in all species. This can be used as a baseline for suture more comprehensive studies.

Ultimately, the complexity in shape and magnitude of the coral reflectance depends on the variability of the spectral absorption and fluorescence properties of not only the endosymbiotic dinoflagellate pigments residing within the coral colony but also on the ectodermal and endodermal host tissues [5]. The reduced prediction effectiveness shown in Fig 7 might reflect the influence of host tissues on the coral’s Rrs. Further, the sources of additional pigments (from endolithic algae or other organisms) may not necessarily be close to the coral’s surface and, hence, their influence on the Rrs may be negligible, therefore contributing to the reduction on its effectiveness to predict pigment concentration. Additionally, a number of factors related to the skeletal structure (i.e., differences in skeletal density, arrangement of the skeletal crystals, colony form, etc.) affect the light scattering within the colony [43] and, fundamentally, the spectral reflectance properties of the coral holobiont. Next steps for this work are to demonstrate the use of Rrs as a technique that can easily be applied to estimate coral pigment conditions through time to identify any temporal variability corals may exhibit under different stress conditions (thermal, turbidity, nutrients) under changing climate and anthropogenic effects. Further, we propose the addition of more samples from each of the species studied from Puerto Rico and other Caribbean sites as well as a thorough genetic analysis of their respective symbiont clades to reduce the uncertainty on the relationship between pigment concentrations and the presence or absence of specific symbiont clades (or community of clades) within particular coral species.

## Supporting Information

S1 Table Percent of colonies from each species containing a particular pigment. (XLSX)
